# Positive Outcome in Catastrophic Momordica charantia-Associated Herb-Induced Liver Injury: A Tale of Two Cities - From Gonaives, Haiti to New York City

**DOI:** 10.7759/cureus.46597

**Published:** 2023-10-06

**Authors:** Ifeoma Kwentoh, Anuoluwapo Adelodun, Theophilus Bortier, Daniel Ogbovoh, Earl Scott

**Affiliations:** 1 Internal Medicine, Columbia University College of Physicians and Surgeons, Harlem Hospital Center, New York, USA; 2 Internal Medicine, Columbia University College of Physicians and Surgeons, Harlem Hospital Center, New york, USA; 3 Psychiatry and Behavioral Sciences, Harlem Hospital Center, New York, USA; 4 Emergency Medicine, Harlem Hospital Center, New York, USA

**Keywords:** national poison control center, momordica charantia, liver injury case report, herbal phytoconstituents, complementary and alternative medicine(cam), public health and safety, toxicology and poisoning, hepatic profile, herb-induced liver injury (hili)

## Abstract

Herb-induced liver injury (HILI) is a global concern due to the uptrend in Complementary and Alternative Medicine (CAM). The authors add to the current literature by reporting a case of a 61-year-old man with recent travel to Haiti. His past medical history include hepatitis C virus treated in 2021 with a sustained virologic response (SVR). He presented with profound weakness and abnormal liver transaminases in the thousands. It was initially unclear what the etiology of the patient’s hepatocellular necrosis was, however, the level of abnormality was most consistent with either toxic metabolic injury or vascular ischemic injury. We initiated N-acetylcysteine and vitamin K and had a positive outcome. Upon further questioning, he admitted to consuming an herbal product cleansing tea called “asowosi” in large quantities. We searched the botanical name of the extract and found the active ingredient was *Momordica charantia*. The team utilized the updated Roussel Uclaf Causality Assessment Method (RUCAM), and the results demonstrated a highly probable relationship with *M. charantia*.

## Introduction

Herb-induced liver injury (HILI) is a component of adverse drug reactions seen with the use of herbal products [1]. The mechanism of HILI is poorly understood; however, most cases have been largely attributed to immune-mediated etiology based on mouse-model studies [2]. The incidence rates of herbal hepatotoxicity are for the most part unknown [3]. A literature search including the keyword phrase plant extract documented approximately 21 herbs between 1966 and 2015 (50 years' worth of reports). Of note the case reports described did not underscore all cases in the literature, with prompting and encouragement for additional case reports welcomed online [4]. The use of herbal and dietary supplements (HDS) worldwide has seen substantial exponential growth; however, the prevalence of Complementary and Alternative Medicine (CAM) remains unknown [5-7]. In the United States alone the out-of-pocket costs were estimated to reach $14.8 billion in 2008, and by 2012 the figures went up to $30.2 billion [8]. The accurate assessment with the modified causality assessment is sensitive and non-specific for making a diagnosis of HILI. However, there are limitations associated with this assessment tool, including the confounding causality assessment scoring system, under-diagnosis and under-reporting of HILI, the complex botanical compounds involved in the products, and finally the additives used in the same herbal products marketed under different trade names [9-11]. These setbacks make the diagnosis and reporting of these cases very challenging, creating more complexities in the implementation of safety measures used to safeguard and protect the ordinary consumer.

## Case presentation

A 61-year-old male with recent travel to Haiti, and a background medical history of hepatitis C virus treated in 2021 using glecaprevir/pibrentasvir (G/P) (Mayvret) achieving sustained virologic response (SVR), hypertension, Congestive heart failure with low ejection fraction in the 35% range and Type 2 Diabetes. He presented to our emergency room (ER) with mild right upper quadrant abdominal pain, jaundice, and reduced urinary output for 1 day. He had just returned from a 10-day trip to Haiti, before being brought into our ER. He referred to consuming a cleansing tea in large quantities but believed that the tea was not the reason for his acute illness, he called the tea “asowosi”. The patient endorsed that the herbal tea was used for bloating, and gut cleansing and was popular within the local community, hence he blew it off and was not forthcoming with the history. His medication history included aspirin 81 mg, atorvastatin 20 mg at bedtime, clopidogrel 75 mg daily, hydralazine 100 mg daily, metolazone 5 mg daily, pantoprazole 40 mg daily, and Aldactone 25 mg daily.

On physical examination, vital signs were normal except for a very low-grade fever of 100.4 Fahrenheit. Further examination revealed a middle-aged lethargic man, awake, alert, and oriented but he had dry mucus membranes and was acutely ill-appearing and toxic looking, icterus and jaundice were observed but there were no stigmata of chronic liver disease. His mental status examination was normal, and no asterixis was seen. The abdomen was mildly distended, with mild right upper quadrant - tenderness, Murphy’s sign was negative, with no guarding, rigidity, or rebound, except for mild hepatomegaly but no splenomegaly. Bowel Sounds were normal. Other physical examination findings were unremarkable.

**Figure 1:**
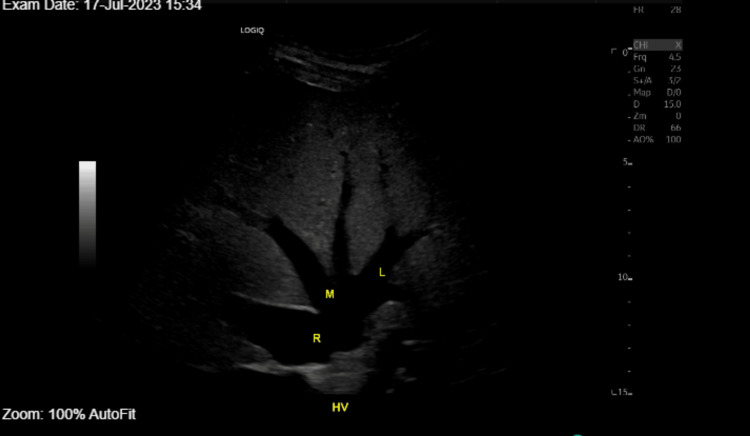
Abdominal liver US with patent IVC, common iliac veins, and hepatic veins, with no evidence of thrombosis. US: ultrasound, IVC: inferior vena cava; HV: hepatic vein; R: right; M: middle; L: left

**Figure 2:**
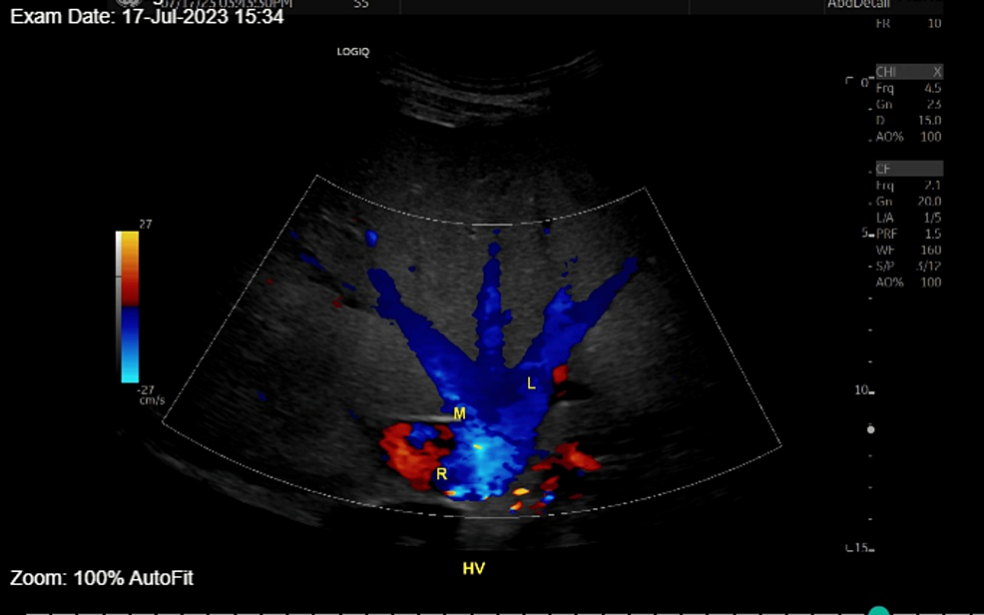
Abdominal ultrasound of the liver demonstrating blood flow in the branches of HV and IVC. Multiple grayscale and color Doppler images of the IVC, common iliac veins, and hepatic veins. HV: hepatic veins; R: Right; M: middle; L: left; IVC: inferior vena cava

Labs at the emergency department showed acute kidney injury on chronic kidney injury with creatinine of 3 from a baseline of 1.3, hyperkalemia, potassium of 6.6, elevated white cell count of 13,000, and elevated creatinine kinase of 544. Blood alcohol and urine toxicology screen were negative. The coagulation profile with prothrombin time and international normalized ratio (INR) were 20.4 and 1.75, respectively. Troponin and brain natriuretic peptide were within normal limits. Lactate of 5.1, total bilirubin was 1.5 up trended from 0.2 with alanine aminotransferase (ALT) 1363, from baseline of 9, aspartate aminotransferase (AST) 2784 from baseline of 28. Blood and sputum cultures were collected. Investigations revealed possible evidence of sepsis (White Blood Count: 13000, procalcitonin: 55.9, lactate of 5.1) and pneumonia (Chest X-ray CXR: basal opacity, no effusion), and acute kidney injury, with hyperkalemia refractory to treatment. Transaminitis with possible shock liver was entertained. The medical intensive care unit (MICU) was consulted due to concerns of sepsis complicated by multi-organ failure. The patient was admitted to the ICU for emergency hemodialysis. Labs at the MICU showed worsening transaminitis. Blood cultures showed no growth after 72 hours and sputum Gram stain demonstrated normal flora. Abdominal ultrasound (US) was done to rule out thrombosis due to possible Budd-Chiari syndrome and to evaluate the inferior vena cava (IVC) and hepatic veins. The scan demonstrated a patent IVC, common iliac veins, and hepatic veins, showing no evidence of thrombosis (Figures 1-2).

The gastroenterology (GI) team was consulted on day 2 of admission as his transaminitis worsened with AST up trending to > 5000 and ALT > 2000. The level of abnormality was most consistent with toxic metabolic injury or vascular ischemic injury to the liver in keeping with the shock liver. Table 1 shows the patient’s prior liver enzymes in the previous admission compared to the recent admission. Since we could not rule out the possibility of toxic injury, we were advised to start N-acetylcysteine. Also following INR and liver enzymes every 8 hours until stabilized (Table 2).

**Table 1:** Patient’s prior liver enzymes in the previous admission compared to the recent admission. R=(ALT/ULN)/(ALP/ULN): alanine aminotransferase/upper limit of normal/alkaline phosphatase/upper limit of normal

Time	ALT (<=41 U/L)	ALP (40 - 129 U/L)	Calculated R-ratio
Prior to admission	9 U/L	88 U/L	0.3
Admission day-1	1363 U/L	157 U/L	27.9
Admission day-5	1,163 U/L	145 U/L	25.8
Day -7 post-admission	60 U/L	82 U/L	2.3
Day -11 post-admission	44 U/L	88 U/L	1.6
1 month post-discharge	11 U/L	104 U/L	0.3

**Table 2:** Liver function test of our patient from Day 1 admission to Day 10 of discharge. ALT: alanine aminotransferase; AST: aspartate aminotransferase; ALP: alkaline phosphatase; PT: prothrombin time; INR: international normalized ratio; NAC: N-acetylcysteine

Lab	Reference Range	Day-1 admission	Day-2 admission	Day-3 admission Vit K and NAC started	Day 5-6 admission	Hosp discharge Day 10
ALT	<=41 U/L	1,363	1,954	1,549	1,163	60
AST	<=40 U/L	2,784	5,520	2,114	856	88
ALP	40 - 129 U/L	157	158	145	136	125
Total bilirubin	<=1.2 mg/dL	1.5	1.9	1.7	1.2	0.7
PT	9.4 - 12.5 second(s)	20.4	25.4	15.7	13.5	12.2
INR	0.88 - 1.12 ratio	1.75	2.17	1.35	1.66	1.11
Total protein	6.4 - 8.3 g/dL	6.5	5.7	5.9	6.1	6.5

GI recommended blood work for autoimmune workup (Table 3) and unusual hepatitis viruses panel (Table 4), including HSV, cytomegalovirus (CMV), HIV, and Epstein-Barr virus (EBV), anti-smooth muscle antibody, antinuclear- antibody (ANA), malaria parasite, and ceruloplasmin were all negative [8-16].

**Table 3:** Autoimmune workup. dSDNA: double-stranded DNA; LKM: liver kidney muscle; ANCA: anti-neutrophil cytoplasmic antibody; Ab: antibody

Autoimmune Test Components	Ref Range and Units	Patient Value
Antinuclear Ab (ANA)	<1:80	Negative
Anti-smooth muscle Ab, IFA	<1:20	1:20
Antimitochondrial Ab	<1:20	<1:20
dsDNA Ab	<10 IU/mL	<1
Myeloperoxidase Ab	<1 AI	<0.2
LKM Antibodies	0.0 - 20.0 Units	<20.1
Perinuclear-ANCA Ab	Negative	Negative
Cytoplasmic-ANCA Ab	Negative	Negative
Immunoglobin A	84 - 499 mg/dL	235
Immunoglobulin G	610 - 1660 mg/dL	1438
Immunoglobulin M	35 - 242 mg/dL	128

**Table 4:** Patient’s hepatic viral panel as recommended by the gastroenterology team. HEP CQL: hepatitis C quantitative testing

Hepatitis Panel Components	Ref Range & Units	Patient’s Result
Hepatitis A IgG Ab	Nonreactive	Reactive
Hepatitis A Ab IgM	Nonreactive	Nonreactive
Hepatitis B surface Ag	Nonreactive	Nonreactive
Hepatitis B surface Ab	Nonreactive	Nonreactive
Hepatitis B core Ab total	Nonreactive	Nonreactive
Hepatitis C Ab	Nonreactive	Nonreactive
Hepatitis C RNA Quant	Target not detected	Target not detected
HEPCQL RE	Not detected	Not detected

**Figure 3:**
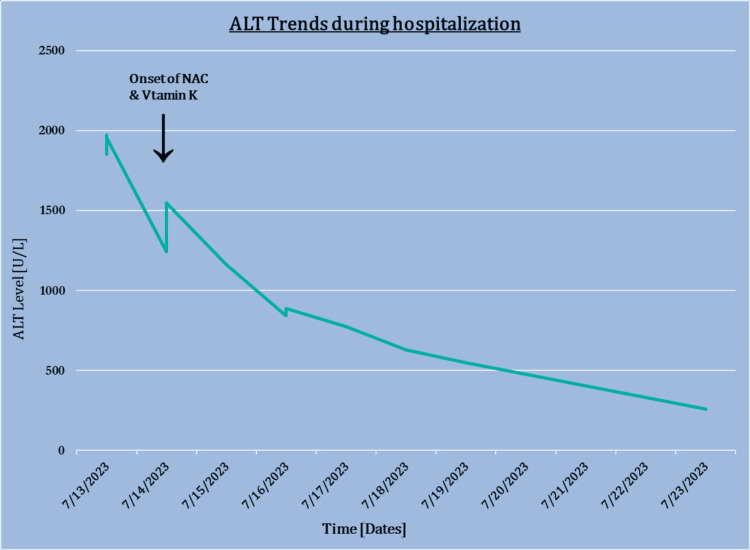
A graph demonstrating ALT levels and timeline from admission to starting NAC. y-axis demonstrates alanine transaminase (ALT) levels and the x-axis shows a timeline from admission to N-acetylcysteine (NAC) and Vitamin K administration.

He received vitamin K 10 mg intravenously (IV) daily for 3 days with a remarkable improvement. N-acetylcysteine was started, and liver enzymes were trended. The patient was worked up with the initial calculation of the R factor which demonstrated R> 5 highly indicative of hepatocellular injury as suspected. ALT trended down following N-acetylcysteine initiation as seen in Figure 3.

**Figure 4:**
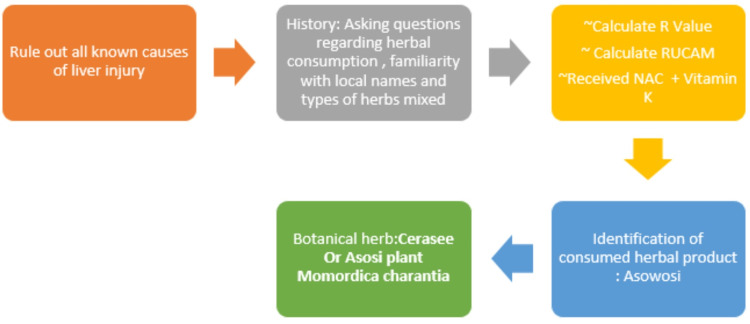
Workflow sheet for the initial assessment of HILI in our patient. HILI: herb-induced liver injury; RUCAM: Roussel Uclaf Causality Assessment Method Step One: Calculation of the R-Ratio: ALT value 1363/upper limit of lab-normal 41 = 34.075 ALP value 157/upper limit of lab-normal 129 = 1.2171 R > 5 (27.99) clearly hepatocellular injury pattern. Step Two: Calculate RUCAM: using a standard table of RUCAM causality assessment = 9 score

Poison control was contacted. One would argue that the liver panel reflected either toxic metabolic injury versus vascular ischemia especially in the setting of low ejection fraction, but cardiology confirmed he had stable coronary artery disease and heart failure with no elevated natriuretic peptide. Our patient had a history of percutaneous intervention (PCI) with stents. Cardiology recommended restarting his dual-antiplatelet therapy due to the risk of stent thrombosis. Infectious etiologies were also considered. All other potential liver insults were considered and ruled out by further history and utilization of the (RUCAM) quantitatively to assess causality in cases of suspected drug-induced liver injury (DILI) and HILI as depicted in Figure 4.

RUCAM assessment (Table 5) shows that there is a high time of onset, course-change on ALT levels with the exclusion of other causes of hepatotoxicity. The total score was deducted as shown. “Highly probable” (score > 8), “probable” (score 6-8), “possible” (score 3-5), “unlikely” (score 1-2), and “excluded” (score ≤ 0). Our patient had a score of 9 which was highly probable.

**Table 5:** Detailed updated RUCAM score of our patient (hepatocellular injury). RUCAM: Roussel Uclaf Causality Assessment Method; ALT: alanine aminotransferase; ALP: alkaline phosphatase; ULN: upper limit of normal.

	Asowosi tea (*Momordica charantia*)
R=(ALT/ULN)/ (ALP/ULN)	27.9
Time to onset	+3
Course of the reaction	+3
Risk factors	+1
Concomitant drug use	0
Search for non-drug cases	+2
Previous information on hepatotoxicity of the drug	0
Response to re-administration	0
Total score	9

Following NAC treatment there was a dramatic trending down of enzyme levels, as demonstrated in Figure 3 [16]. Other than GI and Cardiology, the Infectious Disease (ID) team was involved due to an elevated white count of 13,000, mild anemia, and marked elevation in AST ALT elevation in bilirubin. Blood cultures were negative. Sputum Gram stain was notable for many neutrophils, many epithelial cells, and cultures positive for normal oral flora. A review of imaging studies showed patchy bilateral mid and lower lung infiltrates suggestive of chronic congestive heart failure; however, underlying pneumonia could not be ruled out. The patient had acute hepatitis etiology of which is unknown but the viral cause is unlikely based on available lab results. Cefepime 1 g IV daily, doxycycline 100 mg IV every 12 hours, and vancomycin if positive for methicillin-resistant *Staphylococcus aureus* (MRSA) were considered, and based on that it was quite possible that he may have MRSA responsible for the lung infection but not liver infection. As for acute hepatitis, there does not appear to be any evidence of acute hepatitis B C, A, CMV, EBV, or HIV responsible. ID team strongly believed that hepatitis was unlikely to be an infectious agent in this case. Nephrology recommended hemodialysis due to refractory hyperkalemia and was followed as well as other specialties. Acute HCV infection was excluded with suspected HILI by HCV RNA testing. The patient responded well to NAC and vitamin K and liver enzymes trended down. He was downgraded to the general ward from MICU. The patient never required further dialysis and treatment.

## Discussion

**Figure 5:**
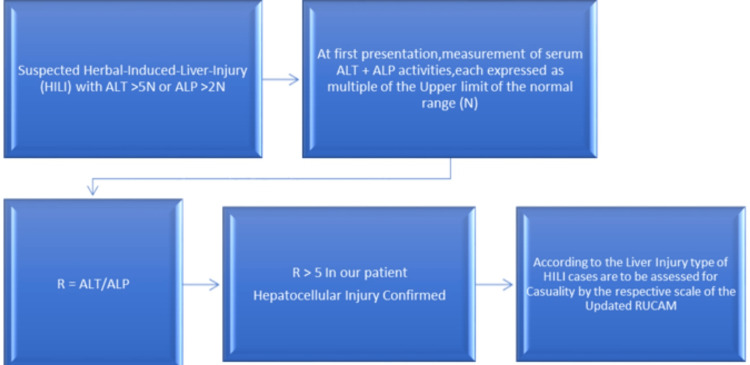
HILI as a top differential, calculation of R-value to determine hepatocellular versus cholestatic injury Step One: Calculation of the R-ratio: ALT value 1363/upper limit of lab-normal 41 = 34.075 ALP value 157/upper limit of lab-normal 129 = 1.2171 R > 5 (27.99) clearly hepatocellular injury pattern. Step Two: Calculate RUCAM: using a standard table of RUCAM causality assessment. HILI: herb-induced liver injury

In this case report, we outline the challenges and methods used to conclude the diagnosis of HILI in our patient which was very complicated and needed a multi-disciplinary team approach. We hope to encourage more succinct and prompt reporting to the database. The initial workflow for suspected HILI should start with a good history and physical examination, obtaining all medications and supplements history from patients, and calculation of the R-value (Figure 5).

The listing of toxic compounds aims to better protect the public and to enhance informed decision-making [12-14]. The profoundly flawed surveillance studies and systematic reviews have the potential to mislead the public, making this diagnosis a very daunting and rigorous process. Indiscriminate use and consumption of herbal products have long been associated with numerous cases of liver injury [15,16]. Many of the cases have required mere supportive management while others have been fatal requiring transplant referral. To date, herbal-induced hepatotoxicity continues to represent an under-reported, poorly understood, and neglected area with poor data acquisition and to a large extent bridge in knowledge [16].

A call for improvement of better data quality of herbal products has long been made. Many clinical and causality assessment tools have been proposed and utilized to better diagnose HILI. Some assessment tools include the scale of the Council for International Organizations of Medical Sciences (CIOMS), Roussel Uclaf Causality Assessment Method (RUCAM), updated RUCAM, and NARANJO et al. proposed a nomogram-like questionnaire designed for adverse drug reaction [16].

A comparative study looking at the accuracy and reproducibility of two scales in causality assessment of unexpected hepatotoxicity was done by Miljkovic et al where they compared the two scales with CIOMS/RUCAM and NARANJO in 19 cases reported during 2004-2009. The conclusion demonstrated that the CIOMS/RUCAM scale showed similar accuracy, but better reproducibility than the NARANJO scale. The study encouraged upgrading and refining of the CIOMS/RUCAM method to better contribute to the detection of unexpected hepatotoxicity [16]. Furthermore, we conducted a literature search for studies associated with *M. charantia*-associated HILI and found none to analyze. NAC may be used off-label, but indications include acute hepatic failure, which was impending in our patient.

## Conclusions

The goal of our case report is to contribute to the database and assist physicians and mid-level providers in becoming increasingly aware of the potential hepatotoxicity of “asowosi” tea to patients with past or current liver injury and to accurately characterize the clinical presentation, and laboratory findings of *Momordica charantia* (asowosi)-induced liver injury. We thought it was interesting that our patient was a Haitian migrant from an indigenous city well known for “asowosi” healing remedies, but he migrated to New York and that brought about an interesting tale between the two cities.
